# Closing the gap: the evolving landscape of integrative oncology education in the United States

**DOI:** 10.3389/fonc.2026.1870175

**Published:** 2026-09-07

**Authors:** Krisstina Gowin, Ashley Larsen, Claudia Witt, Lynda Balneaves, Suzanna Zick, Alissa Huston, Gurdev Parmar, Reya Sharman, Channing Paller, Terri Crudup

**Affiliations:** 1 Cherng Family Center for Integrative Oncology, City of Hope National Medical Center, Irvine, CA, United States; 2 Complementary and Integrative Digital Health Institute of Primary Care – University of Zurich and University Hospital Zurich, Zurich, Switzerland; 3 College of Nursing, University of Manitoba, Winnipeg, MB, Canada; 4 Department of Family Medicine and Nutritional Science, University of Medicine, Ann Arbor, MI, United States; 5 Pluta Integrative Oncology & Wellness Center, Wilmot Cancer Institute, University of Rochester, Rochester, NY, United States; 6 Integrative Health Clinic, Surrey, BC, Canada; 7 University of Arizona Cancer Center, Tucson, AZ, United States; 8 Johns Hopkins School of Medicine, Baltimore, MD, United States; 9 Society for Integrative Oncology, Philadelphia, PA, United States

**Keywords:** hematology oncology, complementary medicine, continuing medical education, fellowship training, integrative oncology, oncology education, integrative medicine, workforce development

## Abstract

Integrative oncology (IO), an evidence informed field that uses mind-body practices, natural products, and lifestyle modification alongside conventional cancer care, has entered a new phase of educational development. The Society for Integrative Oncology–American Society of Clinical Oncology joint clinical practice guidelines on pain, anxiety/depression, and fatigue have sharpened the need for an oncology workforce prepared to counsel patients on evidence-based integrative therapies. Historically, formal integrative oncology-specific training has been sparse, as seen in a published national needs assessment citing that 85.4% of oncology physicians, fellows, and program directors had little or no prior training in integrative medicine in 2022. However, this educational landscape has begun to change rapidly. The Academic Consortium for Integrative Medicine and Health now recognize oncology-specific fellowships, with two board eligible physician fellowship programs now available in the US: the Integrative Medicine in Hematology Oncology fellowship and the Integrative Oncology Scholars Program. Additionally, the (IOS) program offers a multi-disciplinary certificate track. Multiple continuing IO medical education programs have been launched across leading cancer centers, and a comprehensive IO textbook has recently been published. This narrative review maps the current IO educational infrastructure in the United States for physicians, examines competency frameworks and emerging need for curriculum standards for multiple professions, catalogues available resources, identifies persistent barriers to workforce development, and proposes priorities for the field’s next phase.

## 1 Introduction

Approximately half of cancer patients in North America use complementary or integrative therapies during or after treatment, and the utilization rates continue to rise. (1, 2) Integrative oncology (IO) is defined as a patient-centered, evidence-informed field that uses mind-body practices, natural products, and lifestyle modifications alongside conventional cancer treatment (3). Integrative oncology aims to optimize health, quality of life, and clinical outcomes across the care continuum and to empower people to prevent cancer and become active participants, before, during, and beyond cancer treatment. The clinical evidence base has expanded in recent years. The Society for Integrative Oncology (SIO) and the American Society of Clinical Oncology (ASCO) have jointly published practice guidelines on the management of pain, anxiety and depression, and cancer-related fatigue in adults with cancer, providing oncology clinicians with evidence-graded recommendations across common clinical domains (4–6).

Patients seek IO therapies to manage treatment-related symptoms, improve quality of life, address psychological distress, and improve disease outcomes (7). Despite this prevalence, communication about IO therapy use between patients and oncology providers remains limited. Fewer than half of oncologists routinely discuss IO therapy use with their patients, and patients are often unaware of the importance of disclosing IO use to providers (8). Open, informed dialogue between patients and healthcare providers is essential to ensure that IO treatment decisions are guided by rigorous scientific evidence rather than misinformation, anecdotal claims, or unsupported extrapolations.

The clinical consequences are missed opportunities for evidence-based counseling and failure to identify safety consideration, such as potential natural product-drug interactions, thus highlighting the need for clinicians to be better educated in IO and communication strategies.

Few oncology clinicians have had access to formal IO training (9). Until recently, dedicated IO fellowships for physicians, or validated IO curriculum standards, and continuing education (CME) specifically addressing IO issues has been sparse. The educational landscape, however, has changed rapidly in the past decade. This narrative review provides a 2026 update on IO education in the United States (US), mapping available fellowship pathways, CME programs, competency frameworks, and emerging curriculum standards, while identifying persistent workforce gaps and priorities for future development.

## 2 Methods

This narrative review was conducted using a structured search of the published literature. The following electronic databases were searched: PubMed/Medline, Embase, and Google Scholar. Program websites, professional organization resources and government databases were additionally hand-searched to identify literature not captured in peer-reviewed databases.

Search terms were used individually and in combination: “integrative oncology”, “integrative medicine”, “complementary medicine”, “oncology education”, “medical education”, “fellowship training”, “curriculum”, “competency framework”, “continuing medical education”, “continuing professional education”, “workforce development”, “nursing education”, “acupuncture education”, and “advanced practice provider”.

Articles and resources published from January 2000 through May 2026 were eligible for inclusion. The earlier date was selected to capture foundational competency and curriculum development work; the more recent cutoff reflects the rapidly evolving educational landscape documented in this review.

Inclusion criteria were (1): articles, reports, or program descriptions addressing IO or integrative medicine education, training, competency frameworks, or curriculum standards; (2) content focused on or directly applicable to the oncology care context; and (3) published in English. Studies focused exclusively on clinical outcomes of IO interventions without educational components were excluded, as were opinion pieces lacking substantive program or curriculum content. Formal quality appraisal tools were not applied given the narrative scope of this review; however, priority was given to peer-reviewed publications, validated consensus documents, and accreditation standards issued by established professional bodies.

## 3 The IO education gap

A national needs assessment was conducted in 2022 surveying 208 program directors, physicians, fellows, and residents across hematology/oncology, radiation oncology, and palliative care programs in the US. The study found that 85.4% of respondents, including 50.9% of program directors, reported little or no prior training in integrative medicine, documenting a substantial and systemic gap in IO education across the oncology workforce. Despite this deficit, 71.6% of respondents considered IO valuable or essential to patient care, and 64.2% expressed willingness to participate in formal IO training. The primary motivators were perceived patient benefit (73.6%) and patient requests for IO services (68.8%). Regarding preferred training formats, respondents favored certificate programs (48%), and fellowship distinction tracks embedded within existing training (44%) over standalone IO fellowships (9%), suggesting that models integrated into current graduate medical education structures will achieve greater uptake. These findings quantify the mismatch between training exposure and perceived clinical importance that defines the central challenge facing IO workforce development (9).

## 4 Competency frameworks and curriculum standards

Two related but distinct constructs underpin IO education: competency frameworks and curriculum standards. Competency frameworks define what learners must know, be able to do, and demonstrate. These competencies are learner-outcome focused and describe the knowledge, skills, and attitudes required for competent IO practice. Curriculum standards, by contrast, define how programs must be structured: the content that must be delivered, the sequencing and depth of instruction, the delivery methods, assessment approaches, and institutional requirements that guide program design and accreditation. Both are necessary, competencies set the targets, while curriculum standards specify how to get there. The field has made meaningful progress on shared competency frameworks, but the development of profession-specific curriculum standards remains an unfinished task (10).

### 4.1 International IO competency framework

In 2022, Witt and colleagues published the results of a systematic review and international, interprofessional consensus procedure establishing 37 core IO competencies (10). The consensus process involved 28 experts representing seven professions (medical doctors, psychologists, nurses, naturopathic doctors, traditional Chinese medicine practitioners, yoga practitioners, and patient navigators)along with patient advocates, public health experts, and a survey of members of SIO. The resulting framework identified 11 knowledge competencies, 17 skill competencies, and 9 ability competencies. These competencies spanned the assessment of IO therapy evidence, safe integration of modalities with conventional cancer treatment, patient communication, and interprofessional collaboration. This framework is now widely cited as the foundational reference for IO program development internationally.

### 4.2 Fellowship competencies and curriculum standards

The Academic Consortium for Integrative Medicine and Health (ACIMH) functions as both a competency-setting body and a curriculum standards authority for US integrative medicine fellowships. On the competency side, Minichiello and colleagues published updated fellowship core competencies in 2025, substantially expanding the 2014 framework to incorporate health equity principles, trauma-informed care, cultural humility, epistemic inclusivity, clinician well-being, interprofessional collaboration, and planetary health (11, 12). Within this competency document minimum curriculum standards were presented to allow sub-specialty focus, such as in IO, representing another change from the earlier guidelines. The current ACIMH requirement for accreditation specifies the minimum program structure required for fellowship recognition of at least 1, 000 training hours, comprised of: 400 hours of core general IM education, 100 hours of clinical integration, up to 200 hours of research and scholarly activity, and 300 supplemental subspecialty hours. These hours requirements are curriculum standards, and they prescribe what a fellowship program must deliver regardless of institutional context.

## 5 Accredited integrative oncology fellowship

### 5.1 Programs in the US

#### 5.1.1 Integrative medicine in hematology oncology fellowship — University of Arizona/Mayo Clinic Arizona

The Integrative Medicine in Hematology Oncology (IMHO) fellowship, launched in July 2022 as a collaborative program between the University of Arizona Cancer Center and Mayo Clinic Arizona, is the first ACIMH-accredited IO fellowship in the US. The program is designed to run concurrently with a traditional 3-year hematology and medical oncology fellowship, producing clinician-scientists who are board-eligible in both conventional hematology oncology and integrative medicine through the American Board of Integrative Medicine (ABOIM). The theoretical framework and roadmap for this model, grounded in Witt et al.’s 37 core IO competencies have been described by Larsen et al. (13). The program may be a replicable roadmap for institutions developing Accreditation Council of Graduate Medical Education (ACGME) IO fellowship programs. This fellowship program is a hybrid program using the Andrew Weil Foundational Fellowship Curriculum with online modules for curriculum-based learning. Future program models might include dedicated post-graduate (PGY- 7) year integrative tracks, clinical versus research-focused tracks, and certificate pathways for those desiring less intensive training in IO.

#### 5.1.2 Integrative oncology scholars and fellows program — University of Michigan

The Integrative Oncology Scholars (IOS) Program at the University of Michigan, has continued to evolve substantially in recent years. Originally funded through an NCI R25 Cancer Research Education Grant (R25CA203651), the program enrolled its first cohort of 25 scholars in summer 2018 and has since trained more than 100 IO multi-disciplinary professionals from across the US and Canada (14). Significantly, the program has been relaunched and expanded into two distinct pathways. A one-year certificate program remains available to the full interprofessional oncology workforce, including physicians, advanced practice providers (APP), nurses, social workers, pharmacists, and psychologists. A new two-year ACIMH-recognized fellowship track for Doctor of Medicine (MDs) and Doctor of Osteopathic Medicine (DO) has been added, incorporating monthly case conferences, structured support for establishing an IO consult service at the fellow’s home institution, and a second year of general integrative medicine content required for ABOIM board examination eligibility. The program is conducted through an entirely remote model combining team-based learning sessions with eLearning modules. This expansion makes the IOS Program appears to be the only currently identified U.S. IO education pathway that simultaneously trains the full interprofessional oncology team and supports individual physicians in pursuing subspecialty certification.

## 6 Continuing medical education programs in integrative oncology

For the much larger oncology workforce for whom fellowship training is not practical, continuing medical and professional education (CME/CPE) and IO resources represent the most accessible route to building IO competency. High-quality offerings have been developed in recent years, addressing the full spectrum from foundational knowledge to guideline implementation. These programs and resources collectively represent a meaningful expansion from the breadth of information available to the broader oncology workforce (**Tables 1**, **2**).

**Table 1:** Integrative oncology education programs and resources.

Program	Format/audience	URL/notes
IMHO Fellowship (Univ. of Arizona/Mayo Clinic Arizona)	3-yr fellowship for heme/onc fellows. ACIMH-accredited/Board-eligible (ABOIM) at the end of program. Privately funded by the Weil Foundation.	cancercenter.arizona.edu
Integrative Oncology Scholars Program/Fellowship (Univ. of Michigan)	yr certificate (interprofessional); yr ACIMH-accredited fellowship (MD/DO). NCI-funded.	https://medschool.umich.edu/
AWCIM Introduction to Integrative Oncology (Univ. of Arizona)	online CME	https://awcim.arizona.edu/
Mayo Clinic: Integrative Oncology for Healthcare Professionals	Online CME course	https://ce.mayo.edu/
SIO Guideline Training Modules	Free, CME-accredited, interactive. SIO-ASCO guidelines.	integrativeonc.org/training-modules
MSK Fundamentals of Integrative Oncology	Online CME course.	www.mskcc.org
Society for Integrative Oncology Annual Conference	International. CME. Annual. 30+ countries represented.	integrativeonc.org/conference
Comprehensive Integrative Oncology (Zick & Bao, eds.)	Textbook. 5 parts, 61 chapters. Elsevier, 2026. ISBN 9780443301940. Field-defining reference text.	Elsevier; available from major academic booksellers

**Table 2:** Clinical reference resources for integrative oncology practice (2026 update).

Resource	URL/description
About Herbs (Memorial Sloan Kettering Cancer Center)	mskcc.org/aboutherbs — herb-drug interactions, supplement evidence summaries
Cancer Choices	https://cancerchoices.org
Integrative Oncology Toolkit (AWCIM)	cancertoolkit.integrativemedicine.arizona.edu
KNOW Oncology	https://knowintegrativeoncology.org/
NatMed (Natural Medicines Comprehensive) Database	naturalmedicines.therapeuticresearch.com (subscription required)
National Center for Complementary and Integrative Health	nccih.nih.gov
NCI Cancer CAM PDQ Summaries	https://www.cancer.gov/publications/pdq/information-summaries/cam
NIH Office of Dietary Supplements	https://ods.od.nih.gov/factsheets/list-all/
SIO-ASCO Practice Guidelines	integrativeonc.org/practice-guidelines

### 6.1 Andrew Weil Center for Integrative Medicine

The Andrew Weil Center for Integrative Medicine (AWCIM) at the University of Arizona, the same institution that anchors the IMHO fellowship, offers a self-paced online CME course, Introduction to Integrative Oncology. This course is designed for physicians, nurse practitioners, nurses, and physician assistants (15). The curriculum includes the definition and scope of IO; patient use and reasons for pursuing IO therapies; evidence on botanicals, dietary supplements, and natural product-drug interactions; mind-body approaches to cancer supportive care; acupuncture in symptom management; and dietary guidance for patients with cancer. Companion AWCIM courses address Breast Cancer: An Integrative Approach and Nutrition and Cancer. These modules are notable for serving as an academic backbone of the IMHO fellowship’s module based training, demonstrating the scalability of a single, high-quality curriculum across both formal fellowship and self-directed CME contexts.

### 6.2 Mayo Clinic

The Mayo Clinic School of Continuous Professional Development offers Integrative Oncology for Healthcare Professionals, a CME-accredited online course (16). The course is designed to establish foundational IO competency across the Mayo health system and for the wider oncology workforce. Content equips clinicians to counsel patients on IO including lifestyle optimization for cancer outcomes, evidence-based supplement use and safety, cannabis use in the cancer context, and the principles of patient-centered integrative care. Delivered through online modules and recorded presentations. The course offers CME credit for physicians and APPs and is available through June 2028. Its development reflects an institutional commitment at one of the nation’s leading cancer centers to IO workforce development beyond its own clinical programs.

### 6.3 Society for Integrative Oncology guideline-based training modules

The Society for Integrative Oncology (SIO) launched guideline-linked interactive training modules in 2024, funded by a grant from the Scheidel Foundation (freely accessible at integrativeonc.org/training-modules/). The modules review the 2022 SIO-ASCO joint guidelines on Integrative Medicine for Pain Management in Oncology, and the 2023 SIO-ASCO guidelines on Integrative Oncology Care for Anxiety and Depression in Adults with Cancer. Both series are CME-accredited and interactive, designed not merely to transmit knowledge but to support clinicians in implementing guideline recommendations in their practice. Because these modules are free, online, and directly linked to joint SIO-ASCO guidelines, they represent a scalable tool currently available for IO education standardization across practice settings. The development of additional guideline modules (i.e. fatigue) is anticipated by SIO.

### 6.4 Memorial Sloan Kettering

Memorial Sloan Kettering’s (MSK) Integrative Medicine and Wellness Service, one of the longest-established IO programs in the US, offers a suite of online educational resources for clinicians worldwide. The Fundamentals of Integrative Oncology course is a multidisciplinary CME offering that provides oncologists and APPs with evidence-based guidelines for integrating complementary therapies into cancer care (17). Content addresses the physiological mechanisms of non-pharmacologic approaches (mind-body therapies, acupuncture, exercise, and herbal medicine)for cancer-related symptoms including pain, insomnia, nausea, and psychological distress, with practical guidance on natural product-drug interactions and supplement safety. Companion courses include Integrative Oncologyfor Physicians and Fundamentals of Oncology Acupuncture, and MSK’s About Herbs (18) database, which provides an evidence-based reference for natural product-drug interactions for clinical practice.

## 7 The next frontier

### 7.1 Profession-specific curriculum standards

Shared competency frameworks have served an essential catalytic function, demonstrating that consensus on IO learning goals is achievable across professions and providing a common language for program development. But competencies alone are insufficient to drive real-world training at scale. For a framework to be useful for multiple professions it must be translated into curriculum standards: structured specifications of content, sequencing, instructional methods, clinical hours, and assessment tools that are aligned with each profession’s accreditation systems and scope of practice.

The IO workforce now includes APPs, oncology nurses, licensed acupuncturists, pharmacists, social workers, psychologists, physicians, and others with profession specific considerations (i.e. radiation oncology, medical oncology, hematology, surgical oncology, etc.), each operating within distinct educational pipelines and governed by different accrediting bodies. Interprofessional competency frameworks, by design, can only specify what is shared across all professions. They cannot recommend the profession-specific content depth, clinical application, or program structure required for each group. Curriculum standards that do not map onto the structures of individual professions’ training systems are unlikely to be adopted regardless of their conceptual merit. The pharmacy field recognized this early, developing IO curriculum grounded in the pharmacist’s specific role in medication safety and safe supplement use (19). A profession-anchored model for the broader IO field must now be replicate across nursing, APP, clinical psychologists, and other cancer care professionals. In addition, IO curricular standards are also required by complementary medicine practitioners active in cancer care, such as acupuncturists and massage therapists.

### 7.2 Advanced practice providers

The APP oncology workforce is large and rapidly growing. The number of nurse practitioners are projected to grow 35% and physician assistants 20% from 2024–2034, even as oncologist supply growth lags behind rising demand for cancer care (20–22). APPs will increasingly serve as primary oncology care providers for large segments of the patient population, yet no APP-specific IO curriculum standards currently exist. A survey of 556 PAs and NPs at an academic medical center published in 2020, found that APPs generally held positive attitudes toward complementary and integrative medicine but reported limited knowledge of the benefits and risks of specific therapies (23). The authors concluded that a strong IO knowledge base is essential for patient safety and satisfaction, a finding with direct implications for the development of structured IO curriculum standards tailored to APP training programs and accreditation frameworks in this rapidly growing segment of the oncology workforce.

### 7.3 Oncology nursing

Oncology nurses represent a critical interface between patients and integrative care, and the nursing literature has examined attitudes toward the integration of complementary medicine into supportive cancer care, with the majority of those surveyed expressing interest (24). Of the approximately 4.3 million registered nurses in the US, roughly 100, 000 practice in oncology settings (25). A two-round Delphi study engaging 23 experts across 10 European countries recently produced a validated integrative nursing competency profile for nursing students (26), demonstrating that profession-specific curriculum standards for nurses are both achievable and adoptable within existing nursing education frameworks. Whether comparable oncology-specific US nursing curriculum standards should be developed as a distinct subset, aligned with European accreditation structures, is a question the IO field needs to address.

### 7.4 Acupuncture

Acupuncture is among the most widely offered integrative modalities in oncology settings: more than 70% of NCI-designated cancer centers offer acupuncture services (27), and there are approximately 38, 000 licensed acupuncturists in the US (28), with a considerably larger global workforce (29, 30). Despite this reach, oncology specialization is not formally tracked, and no profession-specific IO curriculum standards have been developed for acupuncturists. A peer-reviewed expert opinion on safe acupuncture practice in cancer care has articulated important clinical safety recommendations (31). However, safety guidance and curriculum standards serve distinct purposes: the former tells practitioners what to avoid; the latter tells training programs what to teach, how to sequence it, and how to assess mastery. The development of oncology-specific curriculum standards for acupuncture training programs, aligned with American College of Acupuncture and Oriental Medicine accreditation requirements, remains an unmet need. Other complementary therapy providers, such as massage therapists and others, could benefit from oncology-specific IO curriculum.

### 7.5 Physicians

Physician IO training has received the most sustained attention in the literature, from medical school curricula through integrative medicine fellowship programs. (11, 12, 32–34) A national survey of US oncologists documented persistent knowledge gaps and variable practice patterns regarding natural products use by patients with cancer (35), underscoring the ongoing relevance of curricular development at the physician level. While integrative medicine curricular elements have been described, there is a paucity of literature for physician-specific IO curriculum. The 2025 redefinition of integrative medicine fellowship competencies with a health equity lens represents a particularly important development, as it expanded the accreditation standards to allow specialization, such as IO training for physicians, but there is no formal guidance on specialty specific curricular standards (11). The American Board of Physician Specialties for Integrative Medicine, as curators of the only national integrative medicine board for physicians in the US, the American Board of Integrative Medicine (ABOIM), set the topic domains and focus for physician training currently (36). Curriculum standards beyond ABOIM topic domains that are specific to IO and other oncology specialties (i.e. radiation oncology, medical oncology, hematology, gynecologic oncology, etc.) would help guide specialty specific program development.

## 8 Integrative oncology educational resources

## 9 Persistent barriers to integrative oncology

### 9.1 Education

Several structural barriers limit the reach and impact of IO education. A national needs assessment of 208 US hematology oncology program directors, physicians, and fellows in 2022 identified four consistently cited obstacles: lack of established curriculum, limited faculty expertise, inadequate funding, and time constraints (9). These barriers appeared across hematology/oncology, radiation oncology, and palliative care, and were echoed by program directors at the same rate as trainees, reflecting systemic challenges in graduate medical education rather than individual gaps.

At the learner level, time remains the most consistently cited obstacle. Oncology fellows and practicing clinicians operate under intense clinical and administrative demands that leave minimal bandwidth for additional educational commitments (37). Online and hybrid formats, adopted across all programs reviewed here, mitigate but do not eliminate this barrier. Protected educational time remains a barrier for most IO CME learners. Notably, respondents in the national needs assessment expressed a strong preference for certificate programs (48%) and fellowship distinction tracks (44%) over stand-alone IO fellowships (9%), suggesting that models integrated into existing training structures will achieve greater uptake than *de novo* programs (9).

At the program level, the scarcity of trained IO faculty limits expansion (38). IO remains a young subspecialty with a relatively small pool of clinician-educators who possess both oncology expertise and formal integrative medicine training. The IMHO fellowship and the IOS program are gradually expanding this pool, but demand for IO education, driven by patient utilization and interest in the SIO-ASCO guidelines is growing faster than faculty supply. Financial sustainability is also a significant concern. For example, IMHO fellowship has relied primarily on philanthropic gifts and grants, which may not be sustainable long-term. Future models might need to explore income streams from clinical activity, fellow self-pay, fellowship endowments, and institutional support.

At the system level, the lack of reimbursement for many integrative therapies creates a fundamental misalignment between educational investment and clinical opportunity (39). Clinicians who complete IO training may find that the therapies they are trained to recommend (acupuncture, massage, mind-body programs) are often not covered by insurance, or are unavailable and/or unaffordable for patients in community settings. This reinforces the geographic and socioeconomic disparities in IO access (40).

## 10 Future directions

The convergence of accredited fellowship programs, competency frameworks, SIO-ASCO clinical guidelines, expanded CME opportunities and IO resources [such as a comprehensive IO textbook (41)] provides a foundation for the next phase of workforce development. Here we identify priorities that merit coordinated attention by the IO field.

Standardized, modular IO core curriculum that is adaptable across medical school electives and physician fellowship training, with profession specific training and CME options, warrants development as a collective effort by the IO field.

Additionally, uniform inclusion of IO content in ACGME core curricula is needed. The publication of SIO-ASCO joint guidelines and the high prevalence of patient integrative therapy use provide justification for incorporating IO competencies into the standard training of all ACGME fellowships with curriculum on cancer care.

Replication of the IMHO ACGME fellowship model across additional oncology subspecialties, including radiation oncology, gynecologic oncology, surgical oncology, transplant fellowships, and others would expand the pipeline of IO-trained clinicians available to serve as faculty and program directors.

This is followed closely by the fourth priority of identifying sustained investment in interprofessional IO education. This education can be modeled on the IOS Program, and is needed to reach nurses, social workers, APPs, pharmacists, patient navigators, acupuncturist, and other professions who are most likely to triage patient questions about integrative therapies in day-to-day practice.

Developing a profession-specific IO curriculum standard for multidisciplinary members of the oncology team and oncology focused integrative professionals is another area of effort. These standards should be developed in partnership with accreditation bodies and professional organizations and could help to structure training programs more than competency frameworks published in the academic literature alone. Delphi consensus methods have proven effective for reaching agreement on IO educational content and can be extended to the curriculum standards level for each profession (26).

Another key opportunity is integrating evidence-based concepts directly into daily clinical care through the electronic health record (EHR). Embedding education tools, such as drug-natural product and drug-laboratory interaction checks, within EHR systems (e.g., Epic) and enhancing them with large language model capabilities would enable real-time education and clinical decision support. These tools can continuously incorporate emerging evidence, effectively turning the EHR into a living platform for ongoing education. In doing so, they help clinicians stay current beyond formal training programs and ensure that every patient benefits from the most up-to-date, integrative, and safe care (26, 42).

## 11 International considerations

The applicability of this review is intentionally focused on the US educational infrastructure, to intentional guide IO educational expansion within US training programs. However, several of the foundational frameworks on which this system rests are explicitly international in provenance. The Witt et al. competency framework was developed through an international, interprofessional consensus process, and the European nursing Delphi study cited here demonstrates that profession-specific curriculum development is achievable across diverse educational systems. This raises an important question: to what extent are the educational models described here transferable beyond US borders?

Many international healthcare systems, including Canada, the United Kingdom, Australia, and much of Western Europe, have structural prerequisites for IO fellowship and continuing education programs that are largely similar. Adaptation of US-developed models is plausible in these settings with appropriate modification for local scope-of-practice regulations and professional licensing frameworks.

An important consideration is how to implement educational programs in low- and middle-income countries, where IO utilization is frequently high yet educational infrastructure for formal IO training is vastly more constrained. Ensuring that IO education advances equitably alongside rising utilization across diverse economic contexts will require investment in scalable, low-resource educational models, such as short-course certifications, mobile learning platforms, and Would Health Organization (WHO)-aligned competency frameworks that account for the overlap between IO and traditional medicine systems already operating within low- and middle-income health systems. The IO education community is encouraged to engage with global health equity frameworks as this field continues to mature internationally.

## 12 Conclusions

There remains a gap between the prevalence of integrative therapy use among cancer patients and the preparedness of the oncology workforce to address it. Closing this gap will require not only the expansion of individual programs but coordinated investment in profession-specific curriculum standards, ACGME curricular integration, continued programmatic development and resource expansion, and sustainable funding for trainee education and integrative services.
